# The New York Journal of Medicine and the Collateral Sciences

**Published:** 1845-08

**Authors:** 


### THE NEW YORK JOURNAL OF MEDICINE AND THE COLLATERAL SCIENCES.

This periodical has already exceeded, in duration, several of its predecessors of the Commercial Metropolis, and bids fair to become a permanent member of the corps. The July number, now before us, commences the third year of its existence, and under the auspices of a new and able editor. The name of Professor Charles A. Lee now graces the title page, and we are sure that its subscribers will require no better guarantee for its future character.

In an address "to the patrons of the Journal," Dr. Lee says he "has reluctantly consented to undertake the editorial supervision of its pages, and in doing so," proceeds "to state, briefly, the principles on which it will hereafter be conducted."

These are just and liberal, and conducted in conformity to them, as we have no doubt it will be while under Dr. L.'s charge, the journal cannot fail to prosper.

